# Retrotransposon: an insight into neurological disorders from perspectives of neurodevelopment and aging

**DOI:** 10.1186/s40035-025-00471-y

**Published:** 2025-03-25

**Authors:** Wenchuan Zhang, Chenxuan Huang, Haiyang Yao, Shangzhi Yang, Zeyidan Jiapaer, Juan Song, Xianli Wang

**Affiliations:** 1 School of Medicine, Shanghai Jiao Tong University, Shanghai, China; 2 Xinjiang Key Laboratory of Biological Resources and Genetic Engineering, College of Life Science & Technology, Xinjiang University, Xinjiang, China; 3 Department of Biochemistry and Molecular Cell Biology, Key Laboratory of Cell Differentiation and Apoptosis of Chinese Ministry of Education, Shanghai Key Laboratory for Tumor Microenvironment and Inflammation, Shanghai Jiao Tong University School of Medicine, Shanghai, China; 4 School of Public Health, Shanghai Jiao Tong University School of Medicine, Shanghai, China

**Keywords:** Retrotransposon, Neurogenesis, Aging, Autism spectrum disorder, Schizophrenia, Alzheimer's disease, Amyotrophic lateral sclerosis, X-linked dystonia-parkinsonism

## Abstract

Neurological disorders present considerable challenges in diagnosis and treatment due to their complex and diverse etiology. Retrotransposons are a type of mobile genetic element that are increasingly revealed to play a role in these diseases. This review provides a detailed overview of recent developments in the study of retrotransposons in neurodevelopment, neuroaging, and neurological diseases. Retrotransposons, including long interspersed nuclear elements-1, Alu, SINE-VNTR-Alu, and endogenous retrovirus, play important regulatory roles in the development and aging of the nervous system. They have also been implicated in the pathological processes of several neurological diseases, including Alzheimer's disease, X-linked dystonia-parkinsonism, amyotrophic lateral sclerosis, autism spectrum disorder, and schizophrenia. Retrotransposons provide a new perspective for understanding the molecular mechanisms underlying neurological diseases and provide insights into diagnostic and therapeutic strategies of these diseases.

## Introduction

Neurological disorders are a broad spectrum of conditions affecting the nervous system, ranging from developmental abnormalities to age-related degenerative diseases. Neurodevelopment encompasses the processes by which the nervous system forms, matures, and evolves, while brain aging refers to the gradual decline and structural changes associated with this process. Both neurodevelopmental abnormalities and age-related neurodegeneration contribute significantly to the onset and progression of neurological disorders [1, 2].

Transposons, also known as mobile genetic elements, have garnered increasing attention in the field of neuroscience research. Initially considered "junk DNA", transposons were historically viewed as genomic parasites with no functional significance. However, recent advances in genomic technologies have unveiled their key roles in shaping genome architecture and regulating gene expression. Of particular interest are retrotransposons, which are a subtype of transposons capable of moving within the genome via an RNA intermediate [3]. Accumulating evidence indicates that retrotransposons, including long interspersed nuclear elements-1 (L1), Alu elements, SINE-VNTR-Alu (SVA) elements, and endogenous retroviruses (ERV), play a role in neurodevelopment, neuroaging, and the pathogenesis of neurological disorders.

This review aims to emphasize the impacts of retrotransposons on neurodevelopment and neuroaging, and explore how they contribute to neurological disorders.

## Overview of retrotransposons

### Concept and classification of retrotransposons

**Fig. 1:**
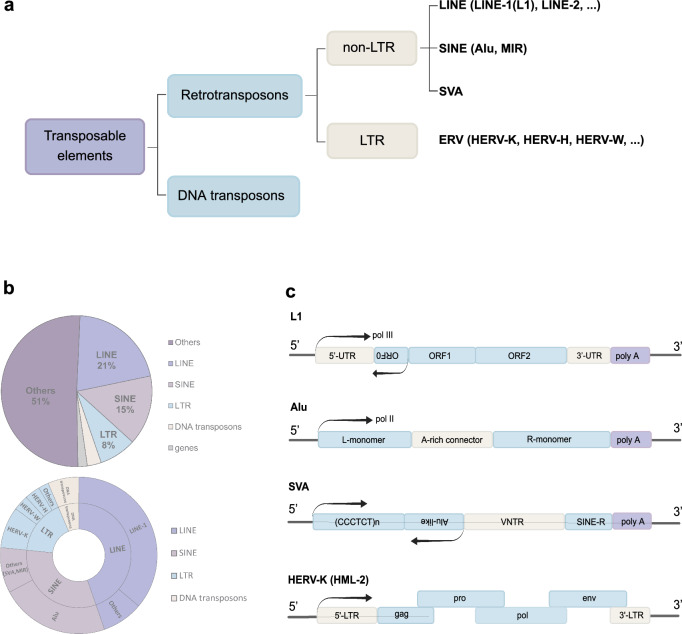
**a** Transposon family classification. Human transposons can be divided into two main groups: retrotransposons (class I) and DNA transposons (class II). Retrotransposons include non-LTR retrotransposons (LINEs, SINEs and SVAs) and LTR retrotransposons. The classification of SVAs is controversial, as they can also be classified as a subfamily of SINEs. **b** The landscape of the human genome and the percentage of each subfamily of retrotransposons. The human genome consists of sequences derived from transposable elements (TE) (47.0%), coding sequences (2.0%), and ‘other’ sequences (promoters, enhancers, introns, non-coding RNAs, telomeres, centromeres, and pseudogenes; 51.0%). TEs are composed of LINEs (44.7%), SINEs (31.9%), LTRs (17.0%) and DNA transposons (6.4%) [46]. The outer circle shows the approximate percentage of subfamilies in each family. To make the graph easier to read, we treat SVAs as a subfamily of SINEs. **c** Structure of retrotransposons. RC-L1s (retrotransposition-capable L1s) contain a 5'-UTR, two internal RNA polymerase II promoters, and three open-reading frames (ORF0, ORF1, ORF2). ORF0 peptide enhances L1 mobility, while ORF1 and ORF2 encode functional proteins for retrotransposition (ORF1p and ORF2p). ORF2p has endonucleases (EN) and reverse transcriptase (RT) activities. The RC-L1s are autonomous because they produce the functional proteins ORF1p and ORF2p required for reverse transcription [10]. The Alu sequence consists of left and right monomers, followed by an adenosine-rich sequence, and transcribes via RNA polymerase III [47]. Sine-VNTR-Alu (SVA) includes a CCCTCT repeat, an Alu-like domain, a VNTR complex, and a poly-A tail. HERV-K (HML-2) contains viral genes (*gag*, *pro*, *pol*, *env*) flanked by two LTRs. Gag is cleaved into structural proteins, while Pol is cleaved into polymerase-active enzymes (RT, RNaseH, IN). Env is spliced into peptides, surface, and transmembrane units [48]

Transposons are mobile genetic elements that account for over 50% of the human genome [4]. They are broadly divided into two categories: retrotransposons (class I transposons) and DNA transposons (class II transposons) (Fig. 1a). Retrotransposons move via a ‘copy-and-paste’ process through reverse transcription of RNA, while DNA transposons move through a ‘cut-and-paste’ mechanism [5]. Although DNA transposons are active in bacteria, archaea, and many eukaryotes, they remain inactive in most mammals, unlike retrotransposons [6].

Retrotransposons are classified into long-terminal repeat (LTR) and non-LTR subclasses based on evolutionary lineage, structural feature, and mechanism of reintegration within the host genome (Fig. 1a). LTR elements, which encompass endogenous retroviruses (ERVs), reinsert through an integration-dependent (IN) pathway [7] and comprise approximately 8% of the human genome (Fig. 1b). Although ERVs are largely inactive in terms of retrotransposition, certain subfamilies, such as HERV-K, remain transcriptionally active and have been implicated in various diseases, as we will discuss later in this review.

In contrast, non-LTR retrotransposons employ a target-primed reverse transcription (TRPT) strategy [8]. These non-LTR elements include both long and short interspersed nuclear elements (LINEs and SINEs), which collectively constitute about 30% of the human genome sequence [9], as well as SVA elements. Subfamilies of these retrotransposons are further categorized based on their sequence conservation and evolutionary age. Notably, the human-specific LINE subfamily L1HS is the only autonomously active and transposable element in the human genome. Although most LINE copies are ancient and inactive due to mutations [9, 10], each genome contains an estimated 80–100 full-length L1HS copies that retain retrotransposition capability [11–13]. Transcription of these elements generates an approximately 6-Kb bicistronic RNA, encoding all the components necessary for successful reintegration into the genome. Other retrotransposons, such as Alu and SVA, are classified as "non-autonomous" because they depend on L1 machinery to facilitate their RNA integration into the genome [10, 14].

In addition to retrotransposition, both young and ancient retrotransposon elements can affect genome structure and function in various ways. For example, they can provide enhancers [15, 16], transcription factor-binding motifs [17–20] and epigenetically dynamic sequences [21–24] that can have an impact on the transcriptional activity of the host locus.

In the following sections, we will provide a detailed examination of the structure (Fig. 1c) and function of major retrotransposon families in the human genome, highlighting their roles in health and disease.

### Effects of retrotransposon activity on the structure and function of the human genome

Retrotransposon activity significantly impacts the structure and function of the human genome. Alu insertions, occurring in approximately 1 of 20 births, as well as L1 and SVA insertions, found in 1 of 100–200 births, contribute to significant genomic polymorphism [25–27]. These insertion events can induce mutations. Somatic transpositions especially during early development may lead to pathological outcomes [28–30]. Beyond insertional mutagenesis, retrotransposon transcription can also result in negative consequences. Transposable element (TE)-derived nucleic acids can trigger innate immune responses [31], and their transcripts can generate non-coding RNAs that affect gene regulation [32–34]. Additionally, TE-derived peptides may exhibit cytotoxicity, further contributing to disease development [35].

Retrotransposons are primarily regulated by epigenetic mechanisms, including DNA methylation and histone modifications. These mechanisms ensure retrotransposon silencing, thereby preventing genomic instability. DNA methylation is maintained by DNA methyltransferases (DNMTs), particularly DNMT1. DNMT1 is expressed in adult neurons and plays a role in maintaining retrotransposon repression in non-dividing cells [36–38]. During early development, DNA methylation patterns are reprogrammed, and retrotransposons are initially silenced by H3K9me3-mediated histone modifications, which are later replaced by stable DNA methylation [39–41]. In pluripotent stem cells, TRIM28 and KRAB-ZFPs form a key complex that ensures the repression of retrotransposons [40, 42]. In neural progenitor cells, retrotransposon silencing involves more complex mechanisms, with certain TEs being regulated by both DNA methylation and histone modifications [43–45].

## Retrotransposons and neurogenesis

### Overview of neurogenesis

Neurogenesis refers to the process by which neural stem cells (NSCs) or neural precursor cells/neural progenitor cells (NPCs) proliferate and differentiate to generate new neurons [49]. In mammals, neurogenesis occurs both during embryonic/perinatal stages (embryonic neurogenesis) and in the adult central nervous system (adult neurogenesis) in two specific regions: the subventricular zone of the lateral ventricles and the subgranular zone of the hippocampal dentate gyrus [50, 51]. While adult neurogenesis shares similarities with embryonic neurogenesis, there are significant differences in proliferation rate, differentiation pattern, and changes within the cellular microenvironment [52]. The intrinsic mechanisms that regulate neurogenesis in these two phases remain incompletely understood. However, studies have shown that retrotransposon activity is likely common to both processes of neurogenesis. Here we will address both aspects in detail.

### Effects of L1 on adult neurogenesis

**Fig. 2:**
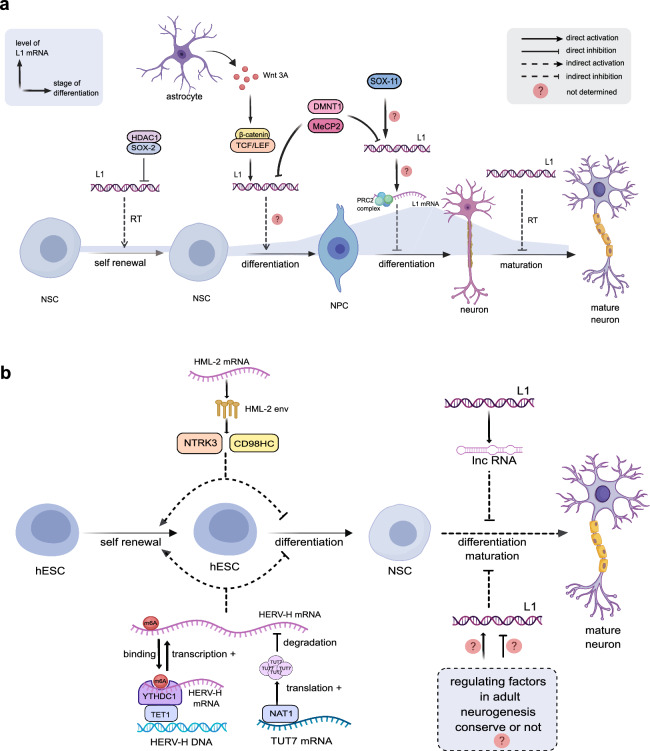
**a** The roles of retrotransposons in adult neurogenesis. L1 mRNA levels are low during NSC self-renewal and gradually increase as NSCs differentiate into neurons [78]. L1 is inhibited by the Sox2/HDAC1 complex formed during NSC self-renewal, and the inhibitory complex is reduced when NSCs differentiate into NPCs [55]. At this time, astrocytes secrete Wnt proteins, which trigger the Wnt-β-catenin signaling pathway in NSCs, in which the downstream signaling molecule TCF/LEF binds to and activates L1, which has been hypothesized to potentially facilitate NSC to NPC differentiation in this process [70–72]. During NPC differentiation into neurons, Sox11 binds to and activates L1 and L1 mRNA is thought to bind to the PRC2 protein, which regulates gene expression in NPCs and inhibits NPC differentiation [75, 76, 79]. Finally, the maturation of neuronal morphology during neuronal maturation is inhibited by L1 retrotransposition [78]. **b** The role of retrotransposons in embryonic neurogenesis. HERV-K (HML-2) expresses Env protein, which interacts with NTRK3 and CD98HC, respectively, and promotes hESC self-renewal and inhibits hESC differentiation [84, 85]. HERV-H RNA also plays a role in promoting hESC self-renewal and inhibiting hESC differentiation. HERV-H RNA is regulated in two ways: (1) NAT1 promotes translation of TUT7 mRNA, and TUT7 promotes degradation of HERV-H RNA [86]; and (2) HERV-H RNA is extensively modified by m6A in hESCs, and YTHDC2 specifically occupies the m6A-modified HERV-H RNA by interacting with the LTR7/HERV-H genomic locus. Subsequently, YTHDC2 recruits TET1 to prevent epigenetic silencing of HERV-H [87]. During NSC differentiation, L1 interacts with lncRNA to inhibit NSC differentiation [88]. It remains to be determined whether the mechanism of adult versus embryonic neurogenesis is conserved during NSC differentiation. RT, retrotranspose

Pluripotent NSCs reside in neurogenic regions of the brain, characterized by three fundamental traits. First, NSCs retain pluripotency and continuously replicate within neurogenic niches. Second, they differentiate into glial progenitors, which mature into astrocytes or oligodendrocytes. Third, these cells can differentiate into NPCs, which can subsequently develop further into neurons [53]. Research has shown notable retrotransposon activity, predominantly L1 activity, during the differentiation phases from NSCs to NPCs, NPCs to neurons, and throughout neuron maturation (Fig. 2a).

Several studies have shown that L1 is expressed dynamically during neural differentiation. Della Valle et al. observed activated L1 during the transdifferentiation of mouse embryonic fibroblasts into dopaminergic neurons [54]. This implies a potential link between L1 activity and neural differentiation. Muotri and Prak et al. demonstrated that L1 retrotransposition occurs at a low but detectable frequency in NSCs, but the frequency is significantly increased during the 48-h period following onset of neuronal differentiation [55, 56]. These studies indicate that L1 is suppressed during NSC self-renewal and activated during their differentiation into NPCs. Furthermore, Jönsson et al. discovered that L1 activation leads to up-regulation of most of the genes involved in neuronal differentiation of human neural progenitor cells (hNPCs). Gene Ontology analysis confirmed that these genes are enriched for synaptic transmission and cellular communication [43]. These findings suggest that L1 plays a regulatory role in the differentiation of NPCs into neurons. L1 can also retrotranspose in mature neurons [30, 57–59]. In conclusion, L1 plays a significant role in adult neurogenesis.

#### Derepression and activation of L1

(a) Epigenetic regulation of L1

DNA methylation is the primary form of epigenetic regulation of L1. DNA methylation leads to repression of L1 transcription, whereas demethylated L1 typically exhibits elevated transcriptional activity [43]. The 5'UTR of L1 harbors an internal RNA polymerase II promoter responsible for directing L1 transcription [60]. Methylation in the 5'UTR and adjacent CpG islands mediates repression of this promoter, thus regulating L1 transcription [61–63]. Studies have demonstrated that the Transcribed-Active (Ta) subfamily of L1, which is the youngest and most active human-specific L1 subfamily, undergoes extensive hypomethylation in pluripotent cells [64, 65] and subsequent methylation during neurodifferentiation [57, 66]. Additionally, Salvador-Palomeque et al. observed highly dynamic L1 promoter methylation during differentiation of human induced pluripotent stem cell (iPSC) into neurons, and the methylation levels increase as the neurons mature. The L1-Ta subfamily and individual L1 promoters exhibit the highest levels of methylation in differentiated neurons, and the lowest levels in hiPSCs and at early stages of neural differentiation [67]. These findings suggest that DNA methylation plays a role in the regulation of L1 during adult neurogenesis. Furthermore, demethylated or hypomethylated sections of evolutionarily young L1 may acquire the histone mark H3K27ac, and then acts as an alternative promoter for numerous nearby protein-coding genes that were previously believed to be associated with neuronal function and psychiatric disorders [43].

DNA methylation is regulated by proteins such as methyl-CpG-binding protein 2 (MeCP2) and DNMT1. Jönsson et al. employed CRISPR-Cas9 to knock down *DNMT1*, a key gene for maintaining DNA methylation, in hNPCs. They discovered a surge in L1 activation upon DNA demethylation in these cells [43]. Moreover, Coufal et al. and Muotri et al. found that MeCP2 modulates L1 retrotransposition during neurodevelopment [66]. MeCP2 binds to methylated DNA in the L1 promoter and suppresses L1 transcription in hNPCs [68].

(b) Regulation of L1 by transcription factors

SOX2 belongs to the SOX-B protein family, which is part of the SOX region Y-associated high mobility group family. SOX2 regulates mammalian embryonic development. It is primarily expressed in early embryonic neuronal cells, and subsequently inhibits the differentiation of NSCs [69]. Muotri et al. discovered that SOX2 binds to HDAC1 to form a repressive complex that interacts with the L1 promoter, suppressing L1 expression in mouse NSCs. As NSCs differentiate into NPCs, SOX2 expression decreases, leading to L1 transcription activation [55]. Kuwabara et al. also observed this phenomenon and identified the overlapping Sox2 and T-cell factor/lymphoid enhancer factor (TCF/LEF)-binding sites (Sox/LEF) in the L1 promoter [70]. Furthermore, bioinformatics analysis has identified numerous L1 elements with identical Sox/LEF double binding sites, including 79 in the human genome, 84 in rats, and 25 in mice [70]. The SOX/LEF site in the L1 promoter is an important inspiration for the subsequent discovery of the Wnt signaling pathway regulating L1 activity.

Besides SOX2, the Wnt signaling pathway also regulates L1 activity. Wnt proteins initiate neurogenesis by acting on a bimolecular switch that includes the SOX/LEF sites within the *Neurod1* promoter [70]. NeuroD1, a basic helix-loop-helix (bHLH) transcription factor in the forebrain, is essential for central nervous system development [71]. During NSC proliferation, SOX2 and HDAC1 are associated on the *Neurod1* promoter and repress it. During NSC differentiation, β-catenin is activated and accumulates in the nucleus in response to astrocyte-derived Wnt proteins [70]. β-catenin forms an activation complex with TCF/LEF, promoting transcription of the *Neurod1* gene [70]. It is worth noting that the SOX/LEF sites have also been identified in the L1 promoter [70, 72], underscoring their crucial role in the differentiation of NSCs into NPCs. It is hypothesized that L1 and *Neurod1* are regulated similarly: during NSC proliferation, the SOX2 and HDAC1 complex suppress the SOX/LEF sites within the L1 promoter. However, during NSC differentiation, Wnt signaling activation increases L1 activity, along with the upregulation of *Neurod1*.

Further research has identified sex-determining region Y-box 11 (Sox11) as an additional key regulator of L1 activity during neuronal differentiation. Sox11, which belongs to the group C of Sox transcription factors, is involved in neurogenesis and tissue remodeling during embryonic development [73]. Sox11 expression is necessary for neuronal protrusion growth and neuronal survival [74]. Previous studies have identified two Sox11-binding sites in the L1 promoter, and overexpression of exogenous Sox11 increases L1 promoter activity [75]. Orqueda et al. found that L1 activity increases during differentiation of induced human SH-SY5Y neuroblastoma cells to neurons, accompanied by increased Sox11 protein binding to the L1 promoter, while Sox11 knockdown inhibits L1 transcription [76]. These findings indicate that Sox11 has increased expression during neuronal differentiation, and binds to the 5'-UTR of L1 and stimulates its transcription. Given the similarity in stemness between human neuroblastoma cells and NPCs, it is hypothesized that Sox11 plays a similar role in NPC differentiation into neurons. However, experimental validation is required to confirm this hypothesis.

#### Roles of L1 in adult neurogenesis

(a) L1 regulates the differentiation of NSCs and NPCs

As previously mentioned, downstream molecules in the Wnt-β–catenin signaling pathway can activate L1 during the differentiation of NSCs into NPCs. Notably, Okamoto et al. discovered a reduction in Wnt3 secretion during aging, which affects the regulation of L1 and results in impaired adult neurogenesis [77]. This suggests that L1 may promote NSC differentiation. It was believed that L1 promotes both NSC and NPC differentiation, as L1 is repressed during NSC division but remains activated post-differentiation. However, recent studies have provided new insights into the regulatory role of activated L1 in NPCs. Toda et al. found that L1 downregulation in mouse NPCs induces precocious neural differentiation, indicating that L1 helps maintain NPCs and slow their differentiation [78]. This suggests that L1 expression plays a critical role in regulating normal neural differentiation, ensuring the formation of unique human brain features [78].

Nevertheless, the molecular mechanisms by which L1 regulates NPC differentiation remain poorly understood. Although effective L1 retrotransposition has been observed in mouse and rat NPCs [55, 58] and abundant L1 transcripts are found in differentiating NPCs, most of these RNAs are not retrotransposed, suggesting that retrotransposition is dispensable in the differentiation of NPCs [78]. It is more likely that L1 plays a role at multiple levels, such as cDNA, RNA, and local transcription. For example, L1 RNAs may regulate NPC differentiation by interacting with Polycomb repression complex 2 (PRC2) [79]. However, the physiological roles of L1 retrotransposition remain largely unknown.

(b) L1 inhibits neuronal morphological maturation

In the study by Toda et al., L1-deficient neurons exhibited significantly increased total dendritic length and higher dendritic complexity [78], suggesting that L1 may inhibit neuronal morphological maturation. Multiple studies have identified L1 retrotransposition in mature neurons. Engineered L1 retrotransposition assay showed that L1 can retrotranspose efficiently in mature neuronal cells [57], indicating that the non-dividing neuronal cells can support extensive L1 mobilization. In addition, most of the somatic L1 insertion occurs at a later stage of neurogenesis [30, 59]. These studies suggest a potential link between L1 retrotransposition in neurons and inhibition of neuronal morphological maturation.

### Effects of retrotransposons on embryonic neurodevelopment and neurogenesis

The process of neurogenesis is basically consistent between embryonic and adult stages [80], although there are subtle differences in the molecular regulation of these stages. For instance, adult NPCs differ significantly from embryonic NPCs in that they remain quiescent for longer periods within the neurogenic niche, while embryonic NPCs are more proliferative [81]. Currently, research on the role of retrotransposons in embryonic neurogenesis remains limited. There are challenges of studying embryonic neurogenesis, including difficulties in acquiring study subjects due to ethical and technical constraints, challenging conditions such as maintaining embryonic neural cells in vitro without losing their physiological relevance, and a lack of direct research methods for tracking retrotransposon activity during specific stages of embryonic neural development. Some studies have been conducted in vitro by inducing embryonic-derived stem cells or indirectly by genealogical tracing to investigate the activity of retrotransposons during embryonic neurodevelopment. Recent development of brain organoid technology has facilitated studies of embryonic neurogenesis. ERVs have been demonstrated to influence embryonic development. Downregulation of ERVs during specific stages of embryogenesis is crucial for ensuring proper developmental progression [82, 83]. The primate-specific ERV isoforms, HERV-H and HERV-K, have been shown to regulate pluripotency and neural differentiation of human embryonic stem cells (hESCs), with evidence suggesting their crucial role in maintaining the transcriptional network required for neural commitment (Fig. 2b).

#### HERVs are activated during stem cell self-renewal and inhibited during neural differentiation

(a) HERV-K

The Env protein of HERV-K (HML-2) preserves the pluripotency of ESCs and impedes neural differentiation. Wang et al. reported that the HML-2 envelope protein is expressed on the cell membrane of stem cells and maintains the stemness through interactions with CD98HC. Conversely, downregulation or epigenetic silencing of HML-2 *env* expression leads to dissociation of stem cell colonies and enhanced neuronal differentiation [85]. Vincendeau et al. demonstrated that prolonged activation of HML-2 in hESCs triggers the classical developmental factor NTRK3, which negatively affects cortical neuronal development [89]. This provides further evidence that the activation of HML-2 inhibits the differentiation of pluripotent stem cells into neurons. Notably, continuous activation of HML-2 in hESCs did not increase stemness, which contrasted with the findings from Wang et al. This discrepancy may be due to the differences in the experimental approaches (activating HML-2 expression using CRISPR engineering [89] versus down-regulating HML-2 expression using siRNAs [85]). In fact, even a mild two-fold overexpression of HERV-K (HML-2) Env in neurons can induce ALS neurotoxicity [35]. Therefore, precise regulation of HML-2 is crucial for maintaining a healthy physiological state.

(b) HERV-H

HERV-H RNA promotes self-renewal of ESCs and inhibits neural differentiation. Takahashi et al. discovered that the NAT1/TUT7/HERV-H axis is crucial in the neural differentiation of pluripotent stem cells: NAT1 enhances TUT7 translation, which in turn promotes the degradation of HERV-H RNA [86]. Additionally, Sun et al. found that HERV-H RNA in hESCs is extensively modified by m6A and that YTHDC2 specifically occupies the LTR7/HERV-H genomic locus. YTHDC2 recruits the DNA 5-methylcytosine (5mC)-demethylase TET1 to prevent HERV-H epigenetic silencing [87]. Functionally, the YTHDC2/LTR7 axis promotes self-renewal of hESCs and inhibits their neural differentiation.

As previously discussed, HERVs undergo tight regulation through various epigenetic modifications during NSC proliferation and differentiation. Aberrant HERV expression may lead to the development of neurodevelopmental tumors, including malignant peripheral nerve sheath tumors (MPNST) [90, 91], Merlin-negative schwannomas [92], meningiomas [92], and atypical teratoid/rhabdoid tumors (AT/RT) [93]. Although these tumors may not directly result from abnormal neurogenesis during embryonic development, they provide a direction for further understanding the epigenetic dysregulation of HERVs and their role in embryonic neurogenesis. Notably, Shah et al. discovered elevated levels of HERV-K transcripts HML-2 and HML-6 in glioblastoma cells. The increased expression of HML-6 is associated with poor patient prognosis [94], while the high level of HML-2 fundamentally supports the glioblastoma stem cell niche. HML-2 maintains glioblastoma stemness and tumorigenesis, transcriptionally activates ESC programs in NPC-derived astrocytes, and alters their 3D cellular morphology by activating the nuclear transcription factor OCT4 [95]. This suggests the potential involvement of HERVs in embryonic neurogenesis.

#### Active L1 inhibits premature NPC differentiation in embryonic neurogenesis

Several L1-fusion transcripts have been detected in various regions of the human brain at different ages, including the cortex, spinal cord, ventral midbrain, mesencephalon, and hindbrain [43]. Evrony et al. detected two somatic L1 insertions in 16 neurons assayed by whole-genome sequencing [28]. Genealogical tracing revealed that one of the events occurred in the developing cortex, while the others may have occurred early in central nervous system development or even earlier [28]. The latter scenario aligns with the embryonic events described in mice [96, 97]. Garza et al. demonstrated the dynamic activity of the L1 promoter in the developing human brain through a multi-omics analysis. LINC01876, a long-stranded noncoding RNA of L1 origin, is a human-specific transcript expressed only during brain development. Silencing LINC01876 by CRISPR interference results in reduced brain organoid volume and premature differentiation of neural progenitor cells [98]. Of note, inhibiting L1 expression in human forebrain organoid results in premature differentiation of NPCs [78]. Similarly, some studies have also found a regulatory role of L1 in NPC differentiation. SIRT6, a deacetylase involved in the regulation of genomic stability, has been shown to suppress L1 activity in the mouse brain [88]. SIRT6 deficiency in NPCs in the embryonic developmental stage of the mouse brain leads to overproliferation of NPCs and delays NPC differentiation [99]. Therefore, embryonic SIRT6 deficiency in the brain during developmental stages may lead to L1 de-repression, resulting in delayed NPC differentiation. Collectively, these findings suggest a physiological role of L1 in protecting NPCs from premature differentiation. There is a growing body of evidence supporting the hypothesis that human-specific traits arise from neoteny, i.e., the finding of slower human neurodevelopment compared to other nonhuman primates [100, 101]. As the expression of L1s in NPCs contributes to the temporal regulation of brain development, it is possible that L1 expression is involved in the emergence of neotenic traits in brain evolution, such as decreased gray-matter volume [102] and slower postsynaptic density maturation [103].

Currently, our understanding of the role of retrotransposons in neurodevelopment is limited. Many questions remain to be answered, such as the physiological significance of the L1 retrotransposon, the specific mechanisms by which L1 influences cell regeneration and differentiation, and whether adult and embryonic neurogenesis share similar molecular regulatory mechanisms. The L1 subfamily has significant research implications. The expression kinetics and regulation vary among L1 subfamily units. For instance, L1MdA_1 and L1MdTf_1 transcripts are highly expressed in NPCs, but the former is downregulated while the latter is further increased in neurons [78]. When using shRNAs to silence L1 universally, it is crucial to consider whether the targeted sequence is conserved across the L1 subfamily. Additionally, HERV-K is expressed during early human development and regulates pluripotency and differentiation of hESCs. However, HERVs play dual roles in neurodevelopment: while their expression is crucial for normal developmental processes, they must be tightly regulated and downregulated at specific stages to ensure proper cellular differentiation. This delicate balance complicates experimental manipulation and makes it challenging to explore their roles in neurodevelopment and disease. The exact physiological impact of HERV-K expression during neurogenesis, and how its dysregulation contributes to developmental abnormalities, remain key areas for future research [83, 85].

Specific retrotransposon subfamilies, such as L1MdA_1, should be a focus in future studies. The repetitive nature of retrotransposons makes it difficult to identify which loci are being transcribed. This problem has been partly resolved with the development of long-read sequencing technologies, which provide higher accuracy in distinguishing repetitive sequences. These advanced sequencing methods, along with models such as organoids, offer new opportunities to investigate the specific roles of retrotransposons in neural development.

## Retrotransposons and aging

Aging is a degenerative process with 12 hallmarks recently identified, including genomic instability, telomere attrition, epigenetic alterations, loss of proteostasis, disabled macroautophagy, dysregulated nutrient-sensing, mitochondrial dysfunction, cellular senescence, stem cell exhaustion, altered intercellular communication, chronic inflammation, and dysbiosis [104]. Aging is a major risk factor for neurodegenerative diseases. Previous studies have found specific changes in the activity of retrotransposons, particularly L1 and HERV-K, during aging.

**Fig. 3:**
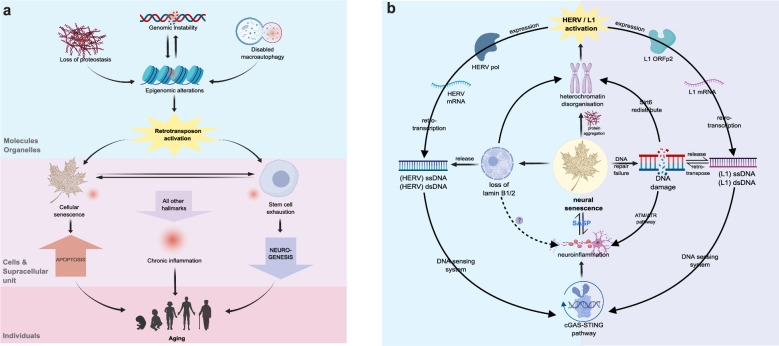
**a** The relationship among retrotransposition activation-associated hallmarks of aging. In the nervous system, some of the hallmarks of aging are associated with the activation of the retrotransposons. Genomic instability and epigenetic alterations are mutually reinforcing, and loss of proteostasis and disabled macroautophagy can also contribute to epigenetic alterations [104]. Epigenetic alterations cause the activation of retrotransposons, leading to cellular senescence and stem cell exhaustion, resulting in increased apoptosis and reduced cellular replenishment, ultimately leading to aging [104]. Additionally, any of the above hallmarks can cause chronic inflammation, which also contributes to aging [104]. **b** The link between retrotransposon activation and neural senescence. Neural senescence leads to the disorganization of heterochromatin and subsequent activation of retrotransposons (L1, HERV) [106, 108, 113, 114]. The activated L1 or HERV forms single- or double-stranded DNA by reverse transcription, which triggers an innate immune response through the cGAS-STING signaling pathway, leading to neuroinflammation and senescence [106, 114]. A senescent neuron can affect adjacent neurons through the senescence-associated secretory phenotype (SASP), leading to further neuronal senescence. Impaired DNA repair in senescent neurons increases DNA damage, leading to SIRT6 displacing heterochromatin to bind at damage sites, exacerbating L1 derepression [88, 105, 107]. Activated L1 integrates into the genome, increasing DNA damage and activating immune pathways. In MERV, senescence is marked by the loss of lamin B1/2, causing chromatin instability and MERV activation, further releasing double-stranded DNA, which triggers the cGAS-STING pathway and ultimately aggravate cellular senescence [106]

Retrotransposon activity within the nervous system is relevant to seven out of the 12 hallmarks of senescence (Table 1). These hallmarks can be divided into two categorie. The first category includes genomic instability, epigenetic alterations, loss of proteostasis, and disabled macroautophagy at the molecular and organelle levels. The second category includes cellular senescence, stem cell exhaustion, and chronic inflammation at the cellular level. Genomic instability, loss of proteostasis, and disabled macroautophagy contribute to epigenetic alterations, which can trigger retrotransposon derepression. This process activates cellular senescence and stem cell exhaustion, leading to increased senescent neurons and a deficiency in neuronal replenishment due to reduced neurogenesis, which in turn contributes to aging. Moreover, the increased retrotransposon activity can cause neuroinflammation, which also contributes to senescence (Fig. 3a). This creates a vicious cycle: senescence de-represses certain retrotransposons, and the activation of retrotransposons reinforces cellular senescence within the nervous system.

**Table 1:** Seven aging hallmarks related to retrotransposition and evidence supporting the correlation

Hallmark category	Retrotransposition-related hallmarks	Evidence	References
Molecules and organelles	Genomic instability	In senescent cells, unstable genomes segregate SIRT6 from L1 loci, reducing L1 inhibition by SIRT6 Loss of B-type lamin revives ERVs, initiating the neuronal aging cascade	[88, 105–107]
	Epigenetic alterations	Loss of H3K9me3 in aged neurons, with cell-type specific heterochromatin loss and L1 activation Aging primate hippocampus shows age-related heterochromatin decondensation and L1 derepression	[108, 109]
	Loss of proteostasis	Tau aggregation in AD brains triggers derepression of L1, SINE, and ERV1, leading to senescence and degeneration TDP-43 aggregation in ALS neurons triggers HERV-K derepression, leading to senescence	[110–112]
	Disabled autophagy	In AD and ALS, impaired autophagy prevents clearance of pathologically aggregated proteins, leading to derepression of L1, SINE, and ERV1, and neurodegeneration Impaired macroautophagy in senescent dopaminergic neurons induces oxidative stress, leading to L1 activation and promoting neuronal senescence	[110–113]
Cells and supracellular units	Cellular senescence	L1 activation in senescent cells causes genomic damage, further aggravating cellular senescence	[113]
	Stem cell exhaustion	Reduction of Wnt3 during aging lowers L1 activity in NSCs, leading to impaired neurogenesis and insufficient neuronal replenishment	[77]
	Chronic inflammation	HERV-K activation in neurons of senescent individuals, AD patients, and ALS patients increases cytoplasmic nucleic acids, triggering inflammation through the cGAS-STING pathway. This leads to neuroinflammation and neuronal senescence Single-nucleus transcriptome mapping of primate hippocampal senescence shows age-dependent genomic and epigenomic changes, one of which is the increased cytoplasmic dsDNA release in the nucleus	[106, 109, 114–116]

### Relationship between L1 and cellular senescence in the nervous system

L1 expression is increased with age in various organisms, including *Drosophila* [117], *S. cerevisiae* [118], *C. elegans *[119], and rodents [105, 107, 120]. Recent studies have linked increased L1 expression and mobility to older age in the mouse brain, particularly in hippocampal neurons [121]. Moreover, Sur et al. used immunohistochemistry to demonstrate that L1 ORF1P expression is elevated in various regions of the post-mortem human brain compared to peripheral tissues such as the kidney, heart, liver, and lung. Notably, their findings also revealed substantial age-related variability in L1 ORF1P expression, particularly in the frontal cortex, suggesting a potential link between L1 activity and aging in the central nervous system [122]. In addition, L1 ORF2P accumulation in the aged primate hippocampal region also suggests a correlation of retrotransposon activation with aging [109]. However, the mechanisms underlying this relationship remain unclear and need further investigation. As extensively demonstrated in cancer research, L1 expression itself can directly lead to cell death through its protein expression [123–125]. Accordingly, a positive feedback is proposed in which cellular senescence triggers L1 activity, potentially exacerbating cellular senescence particularly in neurons, leading to neurodegeneration (Fig. 3b). However, the exact role of L1 in neurodegeneration remains to be fully elucidated, and further research is needed to explore these connections.

#### Neural senescence derepresses L1

In senescent cells, SIRT6, a protein associated with chromatin and involved in DNA repair, is relocated from the L1 locus to DNA damage loci. This results in a reduced inhibition of SIRT6 by L1 [105, 107]. Simon et al. confirmed a similar effect of SIRT6 on L1 in the brain [88]. Moreover, the loss of H3K9me3 in senescent neurons leads to the loss of heterochromatin and subsequent L1 de-repression [108, 113]. Altered expression of factors such as FOXA1, TREX1, and RB1, which epigenetically regulate L1 activity, results in L1 de-repression in senescent cells [105]. Of note, Zhang et al. recently revealed that L1 is activated in senescent neurons of non-human primates. They established the first single-nucleus transcriptomic atlas to demonstrate age-dependent genomic and epigenomic modifications in the non-human primate hippocampus. The alterations are characterized by increased DNA damage, augmented nucleic dsDNA release into the cytoplasm, heterochromatin decondensation, and the resulting L1 de-repression [109].

#### L1 activation induces cellular senescence in the nervous system

Previous studies have shown that L1 expression can induce the cellular senescence phenotype [126]. Recently, Blaudin de Thé et al. reported that acute oxidative stress can induce DNA damage and apoptosis in cultured embryonic midbrain neurons and in adult midbrain dopaminergic neurons in vivo. This often coincides with increased L1 expression. Moreover, enhanced L1 transcription is involved in the damage of H_2_O_2_-induced DNA strand breaks [113]. This suggests that neuronal oxidative stress may increase DNA damage by enhancing L1 transcription, leading to cellular senescence. Additionally, activation of L1 during aging and neurodegeneration may cause DNA damage, resulting in accumulation of neurons with senescence-like traits in different brain regions. The senescence-like state of these cells may propagate to adjacent neurons through SASP (senescence-associated secretory phenotype), leading to chronic neuroinflammation seen in neurodegenerative disorders [127].

Interestingly, Nozawa et al. previously identified that certain types of human cancer cells do not generate telomerase and instead employ the L1 ORF2P protein as an alternate telomere-lengthening mechanism. Inhibiting the activity of L1 ORF2P protein using reverse transcriptase inhibitors results in significant telomere shortening, leading to cessation at the G2 phase of cell cycle and, ultimately, senescence and demise of these cancer cells [128]. This sheds light on the potential telomerase-like functions of L1 ORF2P protein in certain scenarios. Although the role of L1 in telomere elongation in non-cancerous cells has not been proven, these findings suggest that L1 activation may not always lead to cell senescence and provide new insights into its involvement in cellular senescence.

### Neural senescence activates HERV-K, and HERV-K promotes senescence

Elevated expression of ERVs, specifically HERV-K, may be associated with senescence in different species, such as yeast, *Drosophila*, and rodents [129–131]. Similarly, in humans, slightly higher expression of HERV-K (HML-2) on chromosome 1q22 is observed in the peripheral blood mononuclear cells (PBMCs) of older individuals compared to younger individuals [132–134]. Several studies conducted by Zhang et al. and Liu et al. have noted increased expression of HERV-K in senescent neurons of primates, particularly humans [106, 114]. This suggests a correlation between human neuronal senescence and ERV transcriptional upregulation.

The process of HERV-K transcription upregulation in senescent cells has been partly deciphered. Liu et al. discovered increased expression of HERV-K in senescent human cells and various primate tissues [106, 114]. Extracellular HERV-K retroviral-like particles (RVLPs), which are the translation products of HERV-K RNA, induce senescence of young receptor cells through a paracrine effect [106]. The reverse transcription product of HERV-K RNA also stimulates neuroinflammation and neuronal senescence via the cGAS-STING pathway [106, 114]. The study also revealed a relationship between aging-associated neuron-specific nuclear lamina erosion and ERV activation in neurons. Loss of Lamin B1 and Lamin B2, two major structural proteins of the nuclear lamina, leads to epigenetic instability that resurrects ERVs, causing initiation of postmitotic neuronal aging [106]. These studies provide compelling evidence that de-repression of ERVs is closely associated with aging and plays an important role in the mediation of cellular and tissue aging.

Further studies are needed to advance understanding of remaining questions, such as the connection between (epi)genomic instability and increased neuroinflammation in primate hippocampal aging, and the contributions of various modulators of the aging process to pathology.

## Retrotransposons and neurological disorders

Neurological disorders are a significant challenge to the medical field due to their diverse and complex etiology. Extensive research has been conducted to explore the activity of retrotransposons in these diseases. In the following, we will discuss the roles of retrotransposons in neurodevelopment-related diseases including autism spectrum disorder (ASD) and schizophrenia (SCZ), as well as neurodegenerative diseases including Alzheimer's disease (AD), amyotrophic lateral sclerosis (ALS), and X-linked dystonia-parkinsonism (XDP).

### Neurodevelopment-related diseases

Neurodevelopment-related diseases typically involve disruptions in brain development, including rare genetic syndromes, cerebral palsy, congenital neural anomalies, schizophrenia, ASD, attention deficit hyperactivity disorder, and epilepsy [170] (Table 2). Retrotransposons exhibit specific activity in patients with neurodevelopmental disorders, and some retrotransposons play a significant role in the pathogenesis of these disorders.

**Table 2:** Some neurological disorders associated with retrotransposons

Disease	Type of disease	Area of involvement	Genes involved	Related Retro-transposons	Retrotransposon expression/activity and possible pathogenic mechanisms	References
ADHD	Neurodevelopmental disorder	Prefrontal cortex, frontal striatum	*DRD4* *DRD5, *etc	HERV-H	Aberrant RNA expression of the HERV-H *env* observed in PBMCs from ADHD patients	[135, 136]
ASD	Neurodevelopmental disorder	Prefrontal cortex	*POGZ* *TRIM28* *SETDB1*	L1 Alu ERV	Increased L1 expression and reduced epigenetic inhibition in PBMCs from ASD patients; Specific methylation of AluS in patients with mild ASD; POGZ binds to TRIM28/SETDB1 to maintain heterochromatin state and silence ERVs. *POGZ* mutations lead to impairment of the heterochromatin state, resulting in activation of a small subset of ERVs and up-regulation of nearby genes associated with neurological diseases	[137–143]
SCZ	Neurodevelopment- related disease	Prefrontal cortex, frontal cortex	*BDNF* *NTRK2* *DRD2, DRD3* *DISC1*	L1 Alu HERV	Increased L1 somatic insertions and methylation in neurons of schizophrenia patients; Alu insertion into schizophrenia-related loci; Dysregulated expression of SINE B2 in patients with schizophrenia; HERV-W Env proteins are involved in multiple pathways within neurons	[144–147]
Bipolar disorder	Neurodevelopment-related disease	Frontal lobe, basal ganglia, cingulate gyrus, amygdala, hippocampus	*CACNA1C* *ANK3* *VGCC, *etc	L1 HERV-W	L1 hypomethylation in patients Patients positive for HERV-W Env protein have an earlier onset of disease compared to those negative for HERV-W Env protein	[148–150]
Rett Syndrome	Neurodevelopment-related disease	Central nervous system neuron	*MeCP2*	L1	Increased number of L1 insertions and active L1 retrotransposons in the brains of patients	[151]
AT	Neurodevelopment-related disease	Cerebellum	*ATM*	L1Hs	Significantly elevated cerebellar L1 expression in patients; Cerebellar L1 overexpression in mice showing AT symptoms	[152–154]
AD	Neurodegenerative disorder	Cortical neurons, glial cells	*tau*	L1 Alu SVA HERV	Heterochromatin relaxation and active L1 induce G4 accumulation and interfere with gene expression in AD neurons; Alu RNA may induce neuronal apoptosis by activating inflammatory pathways; SVA affects the epigenetic modification of AD-related genes; Tau aggregation induces de-repression of HERVs. Cytoplasmic HERV-derived nucleic acids trigger inflammatory pathways, and glial cells receive and transmit inflammatory signals, releasing neurotoxins that lead to neuronal apoptosis	[112, 155–157]
ALS	Neurodegenerative disorder	Motor neurons, glial cells	*TDP-43*	L1 HERV-K	ALS patients show reduced methylation levels of RC-L1s in neurons, chromatin disorganisation around L1, and increased L1 DNA; TDP-43 cytoplasmic accumulation activates HERV-K, the HERV-K-derived nucleic acids trigger inflammatory pathways, and the glial cells receive and transmit inflammatory signals, release neurotoxins, and cause neuronal apoptosis	[116, 158–160]
CJD	Neurodegenerative disorder	Cerebellum, cerebral cortex	Unknown	HERVs	Increased frequency of HERV detection in the cerebrospinal fluid of sporadic CJD patients	[161, 162]
MS	Neurodegenerative disorder	Central nervous system demyelinated neurons	Unknown	HERVs	Increased HERV expression in brains and cerebrospinal fluid of patients; Increased expression of HERV-W Env in PBMCs from MS patients; 18 HERV families overexpressed in PBMCs from sporadic MS patients	[163, 164]
XDP	Neurodegenerative disorder	Striatal medium spiny neurons (MSN)	*TAF1*	SVA	SVA insertion leads to aberrant expression of TAF-32i in XDP MSNs and exacerbates the aberrant activity of G-quadruplexes by targeting RNA metabolism-related proteins	[165, 166]
AGS	Aging-related disease	Central nervous system neurons, astrocytes	*ADAR* *TREX1, SAMHD1, IFIH1 (MDA5)*	L1 Alu	Mutations in related genes lead to increases in L1- and Alu-derived nucleic acids and the cytoplasmic nucleic acids activate immune-inflammatory responses	[167–169]

*ADHD* attention deficit and hyperactivity disorder, *ASD* autism spectrum disorder, *SCZ* schizophrenia, *AT* ataxia telangiectasia, *AD* Alzheimer’s disease, *ALS* amyotrophic lateral sclerosis, *CJD* Creutzfeldt-Jakob disease, *MS* multiple sclerosis, *XDP* X-linked dystonia-parkinsonism, *AGS* Aicardi-Goutières syndrome

#### ASD

ASD refers to a range of neurodevelopmental disorders characterized by persistent difficulties in social interaction as well as verbal and non-verbal communication, and restricted or repetitive behaviors. The severity of these symptoms varies among individuals [171]. At present, ASD diagnosis is based solely on clinical indications due to the lack of reliable biomarkers [171, 172]. Although the exact causes remain to be fully understood, it is believed that ASD is caused by a combination of genetic predisposition and environmental factors. Immune alterations, particularly maternal immune activation, may be involved in the pathophysiology of ASD [173, 174]. Bioinformatics analyses of disease-related samples suggest that the activity of certain retrotransposons, such as ERV, L1, and Alu, is linked to ASD pathogenesis. Studies of these retrotransposons could potentially reveal novel biomarkers for ASD, thereby improving the diagnostic efficiency.

ERVs have been identified as potential biomarkers for ASD. Specific expression of the HERV family has been detected in the PBMCs of ASD patients [175]. Similarly, two mouse models of ASD have shown significantly increased transcription of the MERV family [176, 177]. Balestrieri et al. found abnormal RNA levels of HEMO (Human endogenous MER34 [medium reiteration-frequency-family-34] ORF), a HERV-encoded protein, in autistic children and their mothers. The study suggests that HERV-H and HEMO could potentially serve as ASD biomarkers due to their high diagnostic performance, as determined through multivariate regression modeling [178]. Notably, the significantly increased expression of the HERV family in mice with VPA challenge on gestational day (GD) 10.5 persisted across F_1_, F_2_, and F_3_ generations in brain and blood. However, the role of HERV in the brains of ASD patients still requires further investigation [176].

The exact role of ERVs in the pathogenesis of ASD may be linked to immunity and epigenetic alterations. ERV overexpression is linked to increased proinflammatory cytokines and Toll-like receptor (TLR) expression, indicating a possible correlation between ERV activity and immune response in ASD [177]. Infection-induced maternal immune activation during pregnancy is a significant risk factor for ASD. Cipriani et al. found dysregulation of ERV and ERV-related gene expression in the prefrontal cortex and hippocampus of mice with prenatal exposure to polyinosinic: polycytidylic acid (Poly I:C) that mimics viral maternal infection [179]. In addition, Tovo et al. found significantly higher expression levels of *TRIM28*, *SETDB1*, and most HERV genes tested in ASD subjects compared to healthy controls, indicating their potential role in ASD pathogenesis [137]. Importantly, *POGZ*, a gene frequently mutated in ASD and other neurodevelopmental disorders [142], plays a key role in maintaining the heterochromatin state by silencing ERVs in ESCs through binding with TRIM28/SETDB1 [180]. POGZ deficiency results in the activation of these ERVs which, in turn, leads to the upregulation of nearby genes associated with neurological diseases [143]. These findings provide potential avenues for exploring the pathogenesis of ASD.

Tangsuwansri et al. analyzed blood samples and found that in ASD patients with severe language disorders, L1 methylation levels are reduced and inversely correlated with the levels of L1 transcript and L1-inserted *C1orf27 *[140]. Interestingly, Shpyleva et al. and Spirito et al. reported divergent findings regarding L1 expression, specifically in the anterior cingulate cortex of ASD patients. This discrepancy may stem from limited sample size and an absence of replication in postmortem brain samples. Future research should take into account the region-specific expression patterns of L1 in the brain, as different brain regions may exhibit distinct levels or regulation of L1 activity. Validating these findings in larger cohorts and across various brain regions will improve the reliability and provide a more comprehensive understanding of L1 expression in ASD.

Current research on Alu has primarily focused on Alu expression profiles and epigenetic modifications, specifically Alu methylation. Blood-based gene expression profiling has revealed differential AluS methylation levels and patterns in specific ASD subgroups but not all ASD individuals, compared to controls. The percentage of the partially methylated pattern ^u^C^m^C is significantly increased in ASD subgroup M (mild), while the percentage of the partially methylated pattern ^m^C^u^C is significantly decreased in ASD subgroup S (savant), compared to controls. Specifically, in the ASD subgroup M, AluS expression is correlated with the methylation status [139]. RNA-sequencing data of the prefrontal cortex tissues of individuals with ASD and normal individuals demonstrated that most of the differentially expressed transposable elements belong to the AluS and AluY subfamilies. Alu elements, though not encoding an open reading frame (ORF), rely on the reverse transcription machinery of L1 for mobilization. Furthermore, the expression levels of Alu elements are correlated with 133 host genes associated with ASD, as well as with cell survival and death of neurons [181]. Although research on Alu is currently limited, these data suggest that Alu elements can provide insights into the etiology and diagnosis of ASD.

#### Schizophrenia

Schizophrenia usually occurs in early adulthood and affects approximately 1% of the population [182]. Specific retrotransposons, HERV and L1, are distinctly modified in individuals with schizophrenia, which may offer diagnostic value.

A growing body of evidence suggests changes in the expression of HERV sequences in schizophrenia patients. Studies have found higher levels of HERV-W-associated *gag* and *pol* transcripts in the peripheral blood of patients with schizophrenia compared to healthy individuals [183–185]. Of note, Karlsson et al. discovered nucleotide sequences similar to the HERV-W *gag* gene in the cerebrospinal fluid of some schizophrenic patients, but not in healthy controls [184]. Furthermore, Tamouza et al. observed HERV-W Env antigenemia in a subtype of schizophrenia, indicating its potential as a marker for subtype classification [186]. These findings suggest a potential association between HERV-W-related proteins, particularly the Env proteins, and schizophrenia.

**Fig. 4:**
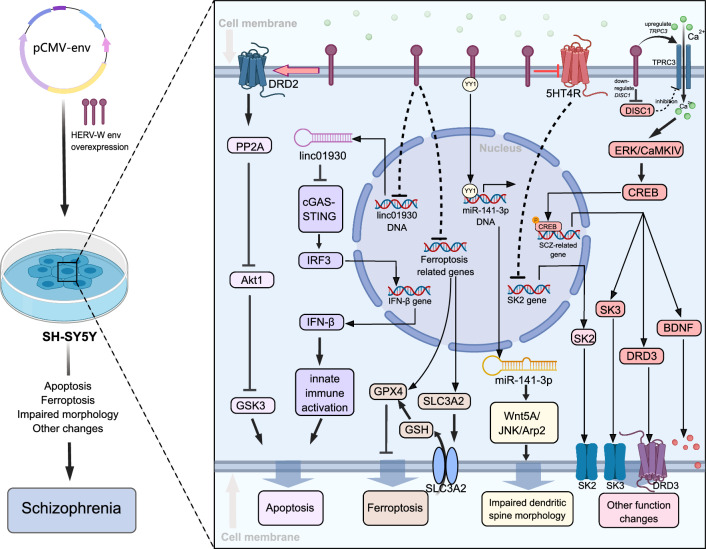
The role of HERV in schizophrenia. Overexpression of HERV-W *env* is widespread in the neurons of patients with schizophrenia. This triggers a series of intracellular signaling pathways that ultimately lead to neuronal apoptosis, ferroptosis, impaired morphology, and other functional changes that cause schizophrenia. The HERV-W Env protein interacts with and activates DRD2, leading to neuronal apoptosis through the DRD2/PP2A/Akt1/GSK3 pathway [144]. In addition, HERV-W Env reduces inhibition of the cGAS-STING pathway by inhibiting linc01930, which induces an immune response and ultimately leads to neuronal apoptosis [187]. HERV-W represses genes involved in ferroptosis repression, leading to an increase in ferroptosis [188]. HERV-W also recruits YY1, which promotes the transcription of miR-141-3p, leading to activation of the Wnt5A/JNK/Arp2 signaling pathway, resulting in neuronal morphological defects [189]. In addition, HERV-W Env inhibits 5HT4R and activates the *SK2* gene [194]. HERV-W Env upregulates the expression of *TRPC3*, leading to an increase in intracellular Ca^2+^ concentration and activation of CREB [190]. This leads to the upregulation of schizophrenia-related genes such as *SK3*, *DRD3*, and *BDNF*, causing functional changes in neurons and ultimately leading to schizophrenia [146, 195, 196]

Recent research has revealed a complex relationship between HERV-W Env proteins and various signaling pathways in neurons (Fig. 4), impacting on neuronal activity and cell fate determination. Huang et al. observed up-regulation of a set of genes related to schizophrenia in human U251 glioma cells, including brain-derived neurotrophic factor (*BDNF*), *NTRK2* (neurotrophic receptor kinase 2), and dopamine receptor D3 (*DRD3*), due to over-expression of HERV-W *env*. This up-regulation is associated with increased phosphorylation of the cAMP-response element binding protein (CREB) [146]. In addition, increased expression of HERV-W *env* in human neuroblastoma cells leads to activation of several signaling pathways, resulting in neuronal apoptosis, ferroptosis, morphological alterations, and changes in other physiological functions. In terms of neural apoptosis, HERV-W env triggers aberrant dopaminergic neuronal processes via DRD2/PP2A/AKT1/GSK3 for schizophrenia risk [144] and promotes antiviral immune response in schizophrenia through the linc01930/cGAS/STING pathway [187], resulting in neuronal apoptosis. Similarly, HERV-W Env induces ferroptosis by degrading GPX4 and SLC3A2 in schizophrenia [188]. In addition, HERV-W Env inhibits the Wnt/c-Jun N-terminal kinases (JNK) non-classical pathway via miR-141-3p, resulting in reduced neuronal density and morphological alterations of dendritic spines [189]. HERV-W *env* expression can also cause other functional alterations. For example, expression of HERV-W *env* leads to an influx of calcium by directly increasing the expression of transient receptor potential cation channel subfamily C member 3 (TRPC3) and decreasing the expression of disrupted-in schizophrenia 1 (*DISC1*) gene, which can also result in activation of TRPC3 [190]. Calcium influx through TRPC3 can activate Erk (extracellular signal-regulated kinase) and CaMKIV (calcium/calmodulin-dependent protein kinase IV). This in turn activates the CREB signaling pathway and affects nerve growth cone induction by BDNF [191–193]. Additionally, Wu et al. discovered that in patients with recent-onset schizophrenia, HERV-W Env is elevated, and the overexpression of HERV-W Env decreases 5-HT4 receptor levels and increases expression of small conductance Ca^2+^-activated type 2 K^+^ channels (SK2) [194]. Overexpression of HERV-W *env* also leads to increased levels of SK3 channels in human neuroblastoma cells [195, 196]. SK2 and SK3 channels are known to regulate neuronal excitability, synaptic transmission, and plasticity. Changes in these channels are associated with the risk of schizophrenia. However, cautions should be paid when interpreting these findings. Most studies have analyzed the overall expression of HERV-W sequences without investigating specific HERV-W loci. Furthermore, the relationship between HERV-W expression and other human diseases remains inconclusive. In addition, little is known about other lineages of HERVs in schizophrenic patients.

Studies of the methylation status and expression levels of L1 in patients with schizophrenia have revealed altered expression of some non-LTR sequences. Bundo et al. discovered increased L1 in neurons from the prefrontal cortex of schizophrenia patients, and L1 insertions in synapse- and schizophrenia-related genes [147]. In addition, over-representation of L1 insertions within the gene ontologies ‘cell projection’ and ‘postsynaptic membrane’ has been identified in the postmortem dorsolateral prefrontal cortex of schizophrenia patients but not of controls [197]. L1 methylation has been detected in peripheral blood leukocytes of individuals with schizophrenia [149, 198, 199]. However, it is important to note that there are significant individual variations in L1 methylation levels. Li et al. reported lower L1 methylation levels in individuals with schizophrenia [149], while another study discovered significantly lower L1 methylation level in patients with first-episode psychosis, paranoid schizophrenia, and methamphetamine-induced paranoia [200].

The SINE family is also associated with schizophrenia. Maternal immune activation, which is a contributing factor to schizophrenia, affects the expression of SINE B2 (a crucial transposable element in the rodent genome) in the stress-sensitive brain regions of rodent offspring [201]. SINE B2 is evolutionarily related to the Alu element in the human genome. An Alu insertion polymorphism (rs71389983) in complete linkage disequilibrium with the schizophrenia GWAS risk variant rs7914558 has been reported, which strongly represses transcriptional activities [145].

At present, there are still many unresolved issues regarding neurodevelopmental diseases, such as the difficulty of early diagnosis and lack of precise treatment. It is important to note that one of the causes of these diseases, including ASD and SCZ, is the malfunctioning of connections between mature neurons and other morphological impairment, resulting in abnormal physiological activities. So, can we turn our attention to neural stem cells, i.e., to find an entry point from the "NSC-NPC-neuron" neurogenesis process, and intervene in the neuronal precursor process, so as to ameliorate the abnormality of neuronal connections? This is the outcome we are striving to achieve.

### Neurodegenerative diseases

Neurodegenerative diseases are conditions characterized by loss of neurons in the brain and spinal cord. Aging is a significant risk factor for these diseases. Of note, retrotransposons play important roles in neuroinflammation and several neurodegenerative diseases.

#### AD

AD is a neurodegenerative disorder characterized by intracellular neurofibrillary tangles of tau proteins and extracellular β-amyloid plaques. Clinical manifestations include progressive cognitive decline, memory loss and language impairment [202]. AD progression is influenced by age, sex, genetic predisposition, and environmental factors, with neuroinflammation being a significant contributor [203]. Retrotransposon activation is involved in the development of AD and may contribute to its progression.

Studies on human postmortem brain tissues and *Drosophila* models revealed that abnormal accumulation of tau protein promotes chromatin relaxation and induces retrotransposon transcription [111]. In line with this, activation of various retrotransposons has been detected by RNA sequencing in human postmortem brain tissues, including L1, Alu, SVA, ERV, and others [111, 155, 156, 204, 205]. Moreover, lamivudine, a reverse transcriptase inhibitor, reduces tau phosphorylation, inflammation, neuronal death, and hippocampal atrophy in transgenic P301S mice [206], further supporting the neuropathological role of retrotransposon activity. Toxic forms of tau protein negatively affect nuclear and genomic architecture in human brain tissue and in various tau protein model systems [207]. The pathogenic tau protein-induced heterochromatin disorganization and subsequent retrotransposon activation are a cause of neuronal degeneration.

In AD patients, retrotransposons such as L1, SVA, and ERV become activated, each performing slightly different roles. Somatic recombination of Alu and L1 elements is widespread in the human genome in normal physiological conditions, with non-allelic homologous recombination being a common feature during double-stranded DNA break repair. However, the somatic recombination profiles are altered in AD [205], which suggests a link between somatic recombination and genomic instability in AD. In addition, accumulation of G-quadruplex structures is observed in AD neurons with relaxed heterochromatin, as well as in normal neurons influenced by chromatin-opening drugs [155]. Of note, the G4 structures found in human neurons are primarily generated by active L1 sequences, which could subsequently disrupt gene expression in AD neurons. Collectively, these studies indicate the involvement of L1 in the pathogenesis of AD. Moreover, knockout of SVA elements at AD-associated risk loci alters the epigenome and the expression of nearby genes. Structurally variable SVAs have the potential to affect the surrounding epigenome and/or transcriptome [156].

Several studies have reported detection of large numbers of ERV transcripts in AD patients or animal models of AD [111, 204, 208]. Ochoa et al. hypothesized that ERV may drive neuroinflammation through toxic intermediates such as double-stranded RNA (dsRNA) based on the similarity of ERV to retroviruses [207]. The levels of dsRNA and dsRNA-sensing machinery are elevated in astrocytes from AD patients, as well as in the brains of tau transgenic mice. This has also been verified in a *Drosophila* model [207]. Moreover, Dembny et al. reported that HERV-K RNA binds to and activates human TLR8 and mouse Tlr7 expressed in neurons and microglia, leading to neurodegeneration [204]. This lays the foundation for understanding the mechanism by which dsRNAs induce immune responses and lead to neurodegeneration. Notably, Evering et al. proposed a putative mechanism for HERV dysregulation in AD: tau protein aggregation induces de-repression of HERVs. The cytoplasmic nucleic acids derived from HERVs activate innate immune sensors, leading to the production of type I interferon and other inflammatory signals. Microglia respond to these signals by releasing cytokines that act on astrocytes. Reactive astrocytes may then release neurotoxins that impair neuronal synapses, ultimately leading to neuronal apoptosis or neurodegeneration. (reviewed in [112]).

The involvement and roles of the SINE family in the pathogenesis of AD are currently a topic of debate. RNA sequencing of postmortem human brains suggests an increase in SINE transcriptional activation [111], leading to the hypothesis that SINEs contribute to AD pathogenesis. Functionally, SINE RNAs have been implicated in β-amyloid pathology in mouse models, where their dysregulation may contribute to disease progression [209]. Although there are no human studies investigating the functions of SINE elements in the brain, Alu RNAs have been shown to trigger inflammatory pathways and apoptosis in non-neural human cells, particularly in retinal pigment epithelial (RPE) cells [157]. Given the shared developmental origin between the retina and neurons, Alu RNAs could potentially play similar roles in neuronal degeneration. Interestingly, sequencing of peripheral blood samples collected from late-onset Alzheimer's disease (LOAD) patients before phenotypic conversion to LOAD, revealed a significant decrease in SINE transcripts, compared to samples collected after phenoconversion [210]. This discovery highlights the intricate regulation of SINEs in AD and calls for further research to clarify their role.

In summary, retrotransposons may be involved in the pathogenesis of AD, with various elements such as L1, Alu, SVA, and ERV potentially playing important roles. However, most existing research has focused on retrotransposon expression in the cortex, with fewer studies exploring other brain regions associated with AD. There are still questions regarding the mechanisms by which retrotransposons cause inflammation in neurodegeneration, and how inflammation leads to differential expression of retrotransposons. Additionally, while over 75 genes have been associated with AD, including risk genes such as *APOE*, *SORL1*, *PICALM*, *TREM2*, and *CR1*, as well as causal genes like *APP*, *PSEN1*, and *PSEN2*, the complete genetic landscape of AD remains not fully defined [211, 212]. Therefore, it is crucial to develop new tools and methods to fully characterize the action sites, preferred sites, and modes of action of retrotransposons.

#### ALS

ALS is a neurodegenerative disease that causes the progressive loss of motor neurons. Patients initially experience muscle weakness, and speech and swallowing disorders, which worsen over time and ultimately lead to fatal paralysis [213]. A hallmark of ALS pathology is the abnormal localization of the trans-activation response (TAR) DNA-binding protein 43 kDa (TDP-43). TDP-43 is typically located in the nuclei of neurons and glial cells and plays a role in RNA regulation. Individuals with ALS demonstrate a loss of TDP-43 in the nucleus and pathological aggregation of TDP-43 in the cytoplasm of motor neurons [214].

ERVs may be involved in the development of ALS, particularly in relation to TDP-43. Several studies have identified increased expression of HERV-K *env* in the postmortem ALS brains [35, 215]. Additionally, there is a significant correlation between the antibody for a specific HERV-K peptide fragment and clinical indicators of disease severity in the serum and cerebrospinal fluid of ALS patients [216]. Moreover, Simula et al. observed a correlation between HERV-K and TDP-43 antibody levels in the serum of ALS patients, and this correlation strengthens with disease progression [217]. Li et al. also observed increased HERV-K transcripts and HERV-K Env protein in ReNcell CX cell-differentiated neurons transfected with TDP-43 [218]. Collectively, these studies suggest a significant association between ALS severity, TDP-43, and HERV-K throughout the onset and progression of ALS.

Furthermore, TDP-43 and HERV-K RNA have been found to co-localize within neurons of ALS patients [215]. TDP-43 directly binds with TE-derived transcripts containing L1, Alu, and ERV sequences. TDP-43 dysfunction leads to reduced association between TDP-43 and TE-derived transcripts, leading to de-repression of the TEs [219]. Importantly, Tam et al. highlighted high levels of retrotransposon expression and TARDBP/TDP-43 malfunction as key ALS characteristics using machine learning techniques. Through RNA knockdown and sequencing technology, they demonstrated that TDP-43 binds to a transpositional segment of the transcript and facilitates in vitro silencing, while pathological TDP-43 aggregation correlates with in vivo transposon de-silencing [110], as confirmed in a *Drosophila* model deficient in TBPH, the TDP-43 homolog [220]. In addition, reactivation of gypsy retrotransposons/ERV is involved in TDP-43 proteinopathy in a *Drosophila* model. Furthermore, the propagation of ERVs can cause DNA damage in nearby neurons, ultimately leading to neuronal death [221]. A recent study further explored the relationship between HERV activation and TDP-43 accumulation. Asparaginase-like-1 protein (ASRGL1) inhibits pathological TDP-43 accumulation by cleaving isoaspartates residues. The *ASRGL1* gene harbors a copy of HML-2. HML-2 RNA induces antisense silencing of *ASRGL1*, interfering with its splicing, translation, and/or activation of the RNA-induced silencing complex, ultimately leading to accumulation of TDP-43 [222]. These findings suggest a mutual reinforcement between ERV expression and TDP-43 proteinopathy, providing insights into the mechanisms of ALS neurodegeneration.

AD and ALS seem to share a similar pathological mechanism: activation of the cGAS-STING pathway by cytoplasmic nucleic acid molecules can trigger an autoimmune response, leading to the initiation of inflammation-associated factors and resulting in neuroinflammation [115, 116]. The cytoplasmic nucleic acids can be from various sources, including TDP-43-mediated mtDNA entry into the cytoplasm [116], dsDNA from reverse transcription of HERV-K, as well as DNA fragmentation caused by inflicted DNA damage in neurons [221]. Subsequently, the activation of the cGAS-STING pathway stimulates TBK1, which in turn activates downstream transcription factors and promotes the expression of related genes [116, 223]. In addition, Romano et al. demonstrated that defects in the siRNA pathway, resulting from dysregulated Dicer-2 activity in TBPH-deficient *Drosophila* neurons, lead to the upregulation of retrotransposable elements (RTE) and subsequent motor neuron degeneration [220], indicating the existence of additional regulatory mechanisms that activate HERV-K in neurons of ALS patients. However, this hypothesis requires further investigation in human neurons.

Previous studies have shown that neurotoxins produced by activated glial cells can cause neuronal death, in addition to the dysregulation of intra-neuronal pathways that leads to neuroinflammation and neuronal demise. Tam et al. identified oxidative and proteotoxic stress, along with glial activation, as key markers of ALS [110], providing a basis for this opinion. In a *Drosophila* model with restricted over-expression of human TDP-43 in glia in subperineurial glia (SPG), Chang and Dubnau found that ERV expression in SPG is required to trigger pTDP-43 signal in neighboring neurons, causing neighboring neuron death, and that silencing ERV in the glial cells can effectively rescue the surrounding neurons [224]. These findings suggest that glial cells may have a more potent capability to induce neuronal death than neuronal dysregulation. However, there is no consensus regarding the neurotoxic mediators of this mechanism. Presently, the HERV-K (HML-2) Env protein is believed to play a key role. Further studies have identified alternative proteins such as the ERVK conotoxin-like protein (CTXLP) [225]. CTXLP, a protein variant produced by a frameshift in the transcription of the HERV-K *env* gene, has demonstrated neurotoxicity primarily in astrocytes of ALS patients.

**Fig. 5:**
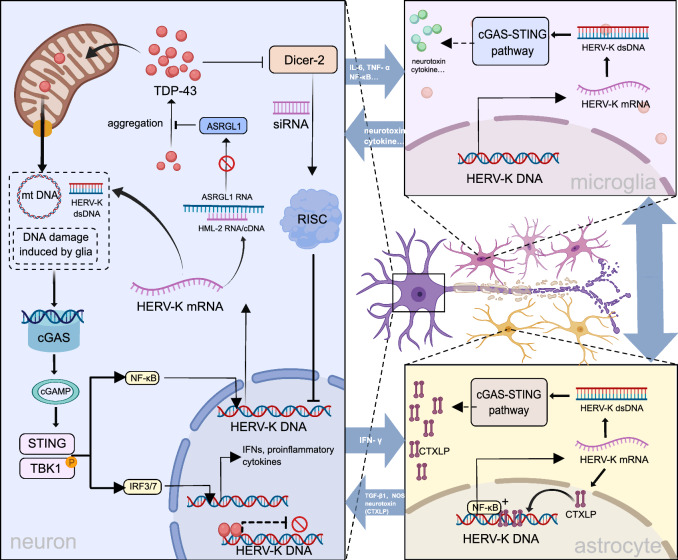
The role of HERV in the pathogenesis of ALS. HERV-K activity is inhibited by nuclear TDP-43. In ALS patients, TDP-43 is deposited in the cytoplasm of neurons and glial cells, including astrocytes and microglia, and HERV-K is derepressed in neurons and glial cells. The cytoplasmic accumulation of TDP-43 can be resolved by ASRGL1; however, HML-2 RNA induces antisense silencing of ASRGL1, thereby leading to the cytoplasmic accumulation of TDP-43 [222]. HERV-K forms dsDNA by reverse transcription, and the dsDNA enters the cytoplasm, activates the cGAS-STING pathway, triggers an immune response, and releases inflammatory factors and other substances that act on neighboring neurons or glial cells, ultimately leading to neuronal apoptosis [115, 220, 221]. TDP-43 deposition in neurons leads to the release of mitochondrial DNA [116], while the toxic effects of glial cells can also lead to the release of DNA fragments from DNA damage [110, 224]. Both events activate the cGAS-STING signaling pathway through cytoplasmic DNA receptors, triggering an innate immune response. In addition, TDP-43 may inhibit the formation of RISC by inhibiting Dicer-2 activity in neurons, thereby reducing the inhibitory effect of RISC on HERV-K [220]. In astrocytes, HERV-K can translate into CTXLP, which has neurotoxic effects [225]

In ALS, inflammation is a significant factor in the disease process. Transcription factors including NF-κB, IRF-1, and IRF-3, play major roles in production of inflammatory mediators [226, 227]. These transcription factors have binding sites on the *TDP-43* promoter, indicating their role in increasing the expression of *TDP-43* and the potential for TDP-43 proteinopathy. Moreover, the LTR regions of HERV-K contain conserved sequences, including two interferon-stimulated response elements. These elements can be activated by type I IFN signaling, leading to the activation of HERV-K expression [228, 229]. Furthermore, experimental evidence has shown that IFN-γ increases the transcription of HERV-K *gag* and *pol*, and enhances activity of reverse transcriptase in astrocyte cell lines [230]. Importantly, CTXLP, which is present in astrocyte cell lines, binds to the interferon-responsive stimulatory element of HERV-K and interacts with NF-κB. Under inflammatory conditions, NF-κB may increase CTXLP expression, induce its migration from the nucleus and aggregation in the cytoplasm, and trigger apoptosis in adjacent neurons [225]. In summary, neuroinflammatory mediators increase TDP-43 expression along with HERV-K. The increased TDP-43 expression can further enhance HERV-K expression, subsequently triggering neuroinflammation, neuron spreading, and ultimately, motor neuron apoptosis (Fig. 5).

Besides HERV, other retrotransposons may also contribute to the development of ALS. The accumulation of TDP-43 is linked to the derepression of retrotransposons such as LINE, SINE, and ERV families [110]. However, their roles seem to be more intricate than initially expected. Some studies suggest that SVA may indirectly contribute to the development of ALS [231, 232], but further research is needed. Regarding L1 elements, studies have shown that some proteins associated with ALS bind to and co-localize with L1 ORF1p ribonucleoprotein particles in cytoplasmic RNA granules [125]. Furthermore, there is a reduction in neuron-specific methylation levels of RC-L1s in ALS patients, along with chromatin disorganization around L1 and an increase in L1 DNA levels [158, 160]. However, there is insufficient evidence to strongly support these findings, and the specific role of L1 in the development of ALS still requires further investigation.

While much progress has been made, the precise mechanisms linking HERV-K to ALS neurodegeneration, as well as its relationship with TDP-43 and neuroinflammation, remain to be fully elucidated. Further studies are needed to advance our understanding of the functional relationship between HERV-K, neuroinflammation, and TDP-43, which may provide insight into therapeutics to slow or halt ALS disease progression.

#### XDP

XDP is an X-linked recessive disorder that typically presents with torsional dystonia, followed by Parkinson's syndrome. The disorder is characterized by neuronal loss and striatal gliosis [233, 234]. Makino et al. identified a disease-specific SVA insertion in an intron of the TATA-Box Binding Associated Factor 1 (*TAF1*) gene. TAF1 is a crucial component of the TFIID pre-initiation complex, which plays a significant role in eukaryotic transcription [235]. Subsequent research, using various genome and transcriptome assembly methods, confirmed the disease-causing mutation to be located at the *TAF1* locus and validated that SVA insertion into the 32nd intron of the gene mediates aberrant transcription associated with XDP [236].

Clinical studies have shown a significant correlation between sequence variants in XDP-specific SVA sequences and phenotypic variations of XDP. Specifically, polymorphic variants within the hexanucleotide repeat region (CCCTCT)_n_ of SVA sequences negatively correlate with the age at onset [237–239] and age at death [239], while positively correlating with disease severity and cognitive impairment [238].

The functional implications of SVA insertion in pathogenesis are uncertain. It is hypothesized that the insertion of SVA affects *TAF1* transcription, as previous studies have demonstrated abnormal *TAF1* exon transcription in the caudate nucleus of XDP patients [235], in cultured XDP fibroblasts [240, 241], and in iPSC-derived NSCs [240]. However, the connection between these transcriptional deficiencies and disease-specific SVA insertions remains unclear. The earlier-mentioned transcription abnormality is characterized by downregulation of the intact *TAF1* transcript, which includes the SVA insertion site [240]. However, patient-derived neural progenitor cells and their extracellular vesicles exhibit higher expression of a TAF1 isoform, TAF1-32i, compared to controls [242]. Similar phenomenon has been noted in iPSCs, NSCs, fibroblasts, and neurons [236]. The generation of this TAF1 isoform, TAF1-32i, is directly linked to the presence of the SVA insertion within intron 32 of the *TAF1* gene. Notably, the level of TAF1-32 is normalized after selective removal of SVA in intron 32 by CRISPR/Cas9 in patient cell lines [236]. These findings support the involvement of SVA insertion in *TAF1* transcription defects.

Furthermore, the mechanism by which the SVA insertion impairs *TAF1* transcription is not fully understood. Bragg et al. conducted a bioinformatics analysis of this SVA sequence and found that multiple motifs, particularly the hexameric repeat structure domain, are predicted to form G-quadruplexes [237]. These G-quadruplexes may cause a delay in *TAF1* transcription by RNA polymerase II. In addition, acetylated histone H3 (AcH3) binding to the exon immediately adjacent to the intronic SVA was decreased in fibroblasts and NSCs derived from XDP, which could be rectified by CRISPR/Cas excision of SVA [166], indicating that SVA insertion contributes to the dysregulation of *TAF1* expression by altering histone status of the insertion region. In another study, Lüth et al. identified a high frequency of CpG methylation in the SVA insertion and surrounding regions using nanopore sequencing. Slight variations in predicted enhancer sites adjacent to the SVA locus were observed between XDP patients and controls [243]. Pozojevic et al. suggested that the XDP-specific SVA reverse transcriptional transposon could act as a transcriptional repressor, inhibiting TAF1 promoter activity [244]. However, Bragg et al. concluded that XDP-specific SVA functions as a promoter [237]. Further research is necessary to clarify the role of SVA in the transcription process.

In conclusion, the role of SVA activity and *TAF1* transcriptional abnormalities in the pathophysiology of XDP remains to be studied. To investigate the impact of *TAF1* mutations on medium spiny neuron (MSN) susceptibility, Tshilenge et al. conducted proteomic examinations on NSCs and induced pluripotent stem cell-derived MSNs obtained from human XDP patients. Functional enrichment analysis revealed a significant representation of pathways related to neurodegenerative diseases, including HD, spinocerebellar ataxia, cellular senescence, mitochondrial function, and RNA-binding metabolism [165]. TAF1, YY1, ATF2, USF1, and MYC, are identified as among the most enriched transcription factors [165]. Notably, an association was found between YY1 and the inherited form of dystonia. Specifically, the SVA insertion causes aberrant expression of TAF-32i in XDP MSNs and amplifies G-tetrasomy dysfunction by targeting RNA metabolism-related proteins, namely SRSF2, POLDIP3, TRA2B, and TIA1. Furthermore, as a repeat expansion disease, XDP exhibits repeat-associated non-AUG (RAN) translation of the (AGAGGG)n-repeat sequences [245], suggesting new avenues for investigating the pathogenic mechanism of XDP.

Future investigations should focus on applying robust computational methods for histological analysis, specifically on the impact of *TAF1* transcriptional abnormality on neurons. Additionally, it is important to confirm the potential regulatory entities such as YY1 and SRSF2.

Currently, studies have identified similarities between functions of the retrotransposons in the natural aging process and those in neurodegenerative diseases. Retrotransposon activation occurs through heterochromatin disorganization and loss of proteostasis during aging. Similarly, retrotransposon activation in a subset of neurodegenerative disorders is caused by aberrant protein aggregation. Additionally, activation of the cGAS-STING pathway after retrotransposon activation is a common feature of both natural aging and some neurodegenerative diseases. Therefore, whether targeting retrotransposons could delay aging in the natural aging process and potentially prevent the onset of neurodegenerative diseases is a promising area for future research.

## Concluding remarks

Retrotransposons are increasingly recognized to play significant roles in neurodevelopment, neuroaging, and neurological diseases. The impact of retrotransposons extends beyond genome insertion. The resulting RNA, DNA, or translation products, particularly those in the ERV family, have pivotal functions. Inserted retrotransposons can potentially modify gene expression or activate functional pathways. These retrotransposons and their derivatives interact with various genes and signaling pathways at different stages, facilitating specific physiological processes such as neural development and aging. Dysregulation of certain molecules in this process could trigger diseases, including neurodevelopmental and neurodegenerative disorders.

However, studies of retrotransposons face several challenges. One significant issue is the repetitive nature of retrotransposons, which complicates accurate mapping and transcript assignment. Studies using human samples are often limited by statistical power, imprecise sequencing methods, and a lack of single-cell resolution, hindering detailed understanding of retrotransposon behavior at the cellular level. In particular, HERVs pose unique difficulties: while their expression is necessary for normal development, they must be tightly regulated and downregulated at specific stages to allow proper cellular differentiation. This dual role complicates experimental manipulation and poses a challenge for understanding their contribution to neurodevelopment and disease [83, 85].

Recent advances in technology provide promising opportunities to address these challenges. Long-read sequencing technologies enable more accurate mapping of retrotransposon insertions, while CRISPR-based gene editing allows precise control of retrotransposon activity in experimental settings. Spatial sequencing techniques are also expected to play a key role in identifying the specific cell types in diseased tissues where retrotransposons are active, helping to clarify their contribution to disease phenotypes. Furthermore, advanced bioinformatics tools and machine learning algorithms can enhance single-cell analysis and provide a deeper understanding of retrotransposon regulation [246, 247]. These approaches, combined with human-based models such as organoids and senescence-induced neurons, will improve the accuracy and relevance of research findings [248, 249]. By utilizing these technological advancements, future studies may identify novel diagnostic and therapeutic targets for neurodevelopmental and neurodegenerative disorders.

## Data Availability

Not applicable.

## Abbreviations

AD: Alzheimer’s disease
AGS: Aicardi-Goutières syndrome
ALS: Amyotrophic lateral sclerosis
ASD: Autism spectrum disorder
AT: Ataxia telangiectasia
BDNF: Brain-derived neurotrophic factor
bHLH: Basic helix-loop-helix
cGAS-STING: Cyclic GMP–AMP synthase (cGAS)–stimulator of interferon genes (STING)
CJD: Creutzfeldt-Jakob disease
CREB: CAMP-response element binding protein
CTXLP: Conotoxin-like protein
DNMT1: DNA methyltransferase 1
DRD3: Dopamine receptor D3
dUTPase: Uracil deoxyribonucleoside triphosphatase
EN: Endonucleases
ERV: Endogenous retroviruses
HERV: Human endogenous retroviruses
hESCs: Human embryonic stem cells
hNPCs: Human neural progenitor cells
IN: Integration-dependent
iPSC: Induced pluripotent stem cell
JNK: C-Jun N-terminal kinases
L1: Long interspersed nuclear elements-1
LOAD: Late-onset Alzheimer's disease
LTR: Long terminal repeat
MeCP2: Methylated CpG-binding protein 2
MS: Multiple sclerosis
NPCs: Neural progenitor cells
NSCs: Neural stem cells
PBMCs: Peripheral blood mononuclear cells
PRC2: Polycomb repression complex 2
RAN: Repeat-associated non-AUG
RC-L1s: Retrotransposition-capable L1s
RPE: Retinal pigment epithelial
RT: Reverse transcriptase
RTE: Retrotransposable element
RVLPs: Retroviral-like particles
SCZ: Schizophrenia
SINE: Short interspersed nuclear elements
SK2: Small conductance Ca^2+^-activated type 2 K^+^ channels
SOX-11: Sex-determining region Y frameshift protein 11
SVA: SINE-VNTR-Alu
TAF1: TATA-Box binding associated factor 1
TDP-43: Trans-activation response DNA-binding protein 43 kDa
TE: Transposable element
TLR: Toll-like receptor
TPRT: Target-primed reverse transcription
TRPC3: Transient receptor potential cation channel subfamily C member 3
XDP: X-linked dystonia-parkinsonism
